# An Analysis of the Gut Microbiota of Fifth-Instar Antheraea Pernyi Larvae and a Functional Exploration of a Bacillus Subtilis Strain

**DOI:** 10.3390/insects16040333

**Published:** 2025-03-21

**Authors:** Xin Xu, Yaxin Gao, Shuanghui Ren, Zhongwen Liu, Yongjun Zhang, Zhen Zhang, Yanxian Lian, Xuwei Zhu

**Affiliations:** 1 Henan Sericultural Research Institute, Zhengzhou 450008, China; xuxinsdaul@163.com (X.X.); 18567289660@163.com (Z.L.); zyj19680505@163.com (Y.Z.); 2 Henan Agricultural University College of Veterinary Medicine, Henan Agricultural University, Zhengzhou 450008, China; 17839706382@163.com (Y.G.); rensh9977@163.com (S.R.); 3 College of Food and Bioengineering, Henan University of Animal Husbandry and Economy, Zhengzhou 450008, China; zhangzhen@hnuahe.edu.cn

**Keywords:** *Antheraea pernyi*, intestinal microbiota, 16SrDNA, lignin-degrading bacteria

## Abstract

This experiment investigated the role of the gut microbiota in the growth and development of *Antheraea pernyi*, isolated gut bacteria related to lignin degradation in oak trees, and preliminarily verified their lignin degradation ability, providing a new tool for the field of biodegradation. The changes in the structure of the gut microbial community of the oak silkworm *Antheraea pernyi* at different developmental stages were analyzed via high-throughput sequencing of the 16SrDNA genes of the gut microbiota of fifth-instar larvae. A series of biological analyses were conducted, including amplicon sequence variant (ASV), microbial community diversity, microbial community composition, species difference, and evolutionary analyses. *Ralstonia*, *Achromobacter*, *Pseudomonas*, *Cupriavidus*, *Bacteroidetes*, *Enterococcus*, and *Bacillus* were the dominant bacterial groups in the gut microbiota of the larvae, with *Ralstonia* comprising the highest proportion, thereby providing a reference basis for a deeper understanding of the functions of the gut microbiota in insect growth and development. In this study, we activated and identified the MYZ028 strain isolated from the fifth-instar larvae of *Antheraea pernyi* through molecular biology, and we determined that the strain belonged to Bacillus subtilis. In terms of performance, the strain exhibited a high lignin-degrading ability and could effectively degrade lignin in a short time and simple environment. Due to the complex and difficult nature of degrading the lignin structure, research on the degradation of lignin by composite strains can be explored in the field of biodegradation.

## Simple Summary

To study the structure of the gut microbiota and the changes in the dominant microbiota during different developmental stages of *Antheraea pernyi*, a high-throughput sequencing analysis was performed on the 16SrDNA genes of the gut microbiota in *Antheraea pernyi* of different ages using metagenomics technology. A series of biological analyses were conducted, including ASV, microbial community diversity, microbial community composition, species difference, and phylogenetic tree analyses. Activation, identification, and performance analyses were conducted on a strain of bacillus subtilis, MYZ028, isolated from the fifth-instar larvae of *Antheraea pernyi*, demonstrating its high lignin-degrading ability.

## 1. Introduction

*Antheraea pernyi* is a silk moth in the order Lepidoptera, family Saturniidae. During the growth and development of *A. pernyi*, the structure of the gut microbiota of the larvae is influenced by various factors. The digestive system of the larvae is relatively simple, with a cylindrical-shaped intestine divided into the foregut, midgut, and hindgut [1]. The gut microbiota of *A. pernyi* plays important physiological roles in the host body, being involved in immunity, digestion, nutritional metabolism, and the degradation of toxic substances [2]. The gut microbiota of *A. pernyi* is highly complex and can be extremely variable. Current research focuses on the diversity of the gut microbiota in Lepidopteran insects, their host relationships, and their role in insect resources and conservation.

The relationship between the gut microbiota and the physiological activities of *A. pernyi* has been explored in previous research, and the results indicate that during the growth and development of *A. pernyi*, the larval stage is the most important regarding the interactions with the gut microbiota [3]. The relationship between the gut microbiota and physiological activities of *A. pernyi* is dynamic and varies across developmental stages, rather than following a simple linear trend. For example, during the pupal stage, some bacterial groups such as *Lactobacillus* and *Bacillus* are relatively high in abundance, indicating that they are involved in nutrient metabolism during the pupal stage [4]. However, bacterial groups such as *Bacteroidetes* and *Bacteroideidae* are low in abundance, suggesting a potentially reduced role in metabolism rather than complete non-participation. When using different culture media to isolate the dominant bacterial genera from the gut of fifth-instar *A. pernyi* larvae, it was found that the genera *Bacillus*, *Acinetobacter*, and *Enterobacter* were dominant, among which *Bacillus* was the main bacterial group; however, the dominant bacterial genera in the gut varied among regions [5]. The microbial abundance in the midgut of *A. pernyi* was far lower than that in the foregut or hindgut [6]. Therefore, further research is needed to determine the relationship between the gut microbiota and the physiological activities of the moth.

The use of the 16S rDNA conserved region to design primers to amplify 16S rDNA fragments, followed by second-generation sequencing and a comparison with NCBI databases, is currently the most widely used method for bacterial identification [7,8]. To the best of our knowledge, this study is the first to conduct a metagenomic analysis of the gut microbiota of the fifth-instar larvae of this species at different developmental stages. Here, the gut microbiota of *A. pernyi* was examined via a metagenomic analysis to determine the structure, function, and metabolic pathways affected by the gut microbiota. In addition, new species may exist in the gut microbiota of *A. pernyi*.

## 2. Materials and Methods

### 2.1. Experimental Animals and Samples

*A. pernyi* larvae: Fifth-instar *A. pernyi* larvae provided by the Tussah Breeding Research Office of Henan Sericultural Research Institute were divided into groups of 0, 2, 5, and 8 days of age (corresponding to samples a, b, c, and d, respectively). After dissecting the larvae, the entire midgut and its contents were excised and treated with liquid nitrogen. The samples were then transferred to sterile centrifuge tubes and stored at −80 °C. Ten larvae were dissected in each sample, and sampling was replicated three times at each time point.

Main reagents: Fusion primers were used in PCR. For the PCR protocol, we used TransGenAP221-02: Trans Start Fastpfu DNA Polymerase. A MOBIO Power Fecal DNA Isolation Kit was used for DNA extraction. The reagents used for library construction and machine sequencing were provided by Illumina (Shanghai Meiji Biomedical Technology Co., Ltd. Shanghai, China), and ultrapure water was used to flush the sequencing instrument pipeline. For DNA quality control, 2% agarose gel electrophoresis was employed. Tris HCl buffer solution, TritonX-100 digestive solution, and sodium hydroxide were of reagent grade. An Axy Prep DNA gel recovery kit was used for DNA recovery.

### 2.2. Sequencing and Sequence Processing

Quantification and homogenization were performed after DNA extraction and dilution. The PCR products were quantified using QuantiFluor™ and an ST blue fluorescence quantitative system (Promega).

The concatenation of the sequences was performed using the Flash 8.0 software (Shanghai Meiji Biomedical Technology Co., Ltd.). The concatenated sequences were compared with the existing bacterial database on UNITE, and the sequences with the greatest differences were retained. We then performed sequence annotation and compared and analyzed the original sequencing data with known data from the SILVA database to determine the microbial genera present in the samples.

### 2.3. Construction and Sequencing of the Illumina Library

PCR was used to add the Illumina connector sequence to the outer end of the target region, and a gel recovery kit was used to recover the PCR products. The products were then washed with Tris HCl buffer and subjected to 2% agarose electrophoresis. Sodium hydroxide denaturation was used to produce single-stranded DNA fragments.

Using the DNA fragments as templates, the base sequences fixed in the Illumina chip were used as primers for PCR. After denaturation and heat treatment, a “bridge” modification was obtained via pairing with a neighboring primer sequence. We used PCR to amplify and generate DNA fragments and used the fragments to form a single strand after DNA amplicon linearization. We labeled four different fluorescent markers using recombinant DNA polymerase and dNTP, and only one base was generated per cycle. A series of gene fragments were read by laser scanning the surface of the reaction disk, and the fragments were aggregated in a single reaction. Chemical methods were employed to remove the fluorescent and termination groups, and the viscosity of the 3′ end could be retained, thereby maintaining the polymerization degree of the nucleic acids. The order of the fluorescent signals was obtained by counting the signals collected in each cycle.

### 2.4. Bioinformatic Analysis

We first preprocessed the data obtained from the Illumina HiSeq sequencing platform, including the concatenated data, filtering out the concatenated sequences. The classification of the sequences was based on 97% similarity [5]. This study included six types of analyses: basic results, amplicon sequence variant (ASV) analysis, community diversity, alpha diversity indices, community composition, and species differences. An evolutionary tree was constructed using Mega 7.0.

### 2.5. Bacterial Strain

The LB liquid medium was activated and prepared (NaCl 5 g, tryptone 10 g, and yeast extract 5 g; distilled water was added to a final volume of 1000 mL at pH 7.2; sterilization was conducted at 121 °C for 20 min). The laminar flow hood was turned on in advance, and two small bottles containing 5 mL of LB liquid medium were placed into the hood for UV sterilization for 30 min. The MYZ028 bacterial strain preserved in glycerol was retrieved from the −80 °C freezer and allowed to recover at room temperature. After recovery, the MYZ028 strain was placed into the laminar flow hood, which had been sterilized for 30 min in advance. A pipette was used to take 10 μL of the MYZ028 strain and transfer it into two 5 mL bottles of LB liquid medium that had previously been autoclaved. The bottles were sealed and placed in a 28 °C constant-temperature shaker at 180 r/min for 24 h to perform the first activation of the MYZ028 strain.

The MYZ028 bacterial liquid that had been cultured in the shaker for 24 h was removed, and UV sterilization was performed for 30 min in the laminar flow hood in advance. A pipette was used to take 100 μL of the MYZ028 bacterial liquid and transfer it into 100 mL of LB liquid medium that had previously been autoclaved. The conical flasks were sealed and placed in a 28 °C constant-temperature shaker at 180 r/min for 24 h to perform the secondary activation of the bacterial strain.

### 2.6. Molecular Biological Identification of Bacterial Strains

The PCR amplification of the 16S rDNA primers used to clone the gene library was carried out using the universal primer 27F: (5′-AGAGTTTGATCCTGGCTCAG-3′) as the forward primer and 1492R: (5′-GGTTACCTTGTTACGACTT-3′) as the reverse primer. DNA extraction was performed using a DNA extraction kit, followed by PCR amplification with the forward and reverse primers, and a dd solution without a DNA template was used as a blank control.

Degradation Effect of MYZ028 Bacterial Strain on Straw: To determine whether the MYZ028 bacterial strain has the ability to degrade lignin, a straw biodegradation experiment was conducted. In the experiment, straw was used as the sole carbon source, and 100 mL of LB liquid medium, 2 g of straw, and 100 μL of bacterial liquid were added to a conical flask. In another conical flask, 100 mL of LB liquid medium, 2 g of straw, and 100 μL of distilled water were added as a control. Both conical flasks were placed in a constant-temperature shaker at 28 °C and 180 r/min for 14 days of cultivation. After the cultivation period, the straw was filtered, washed, and dried, and the degradation rate was calculated by weighing the straw.

### 2.7. Analysis of the Degradation Performance of MYZ028 Strain on Straw Aerobic Composting

An aerobic composting experiment was conducted on the rooftop of the Biological Engineering Building. Four containers of the same specifications were selected and filled with straw compost of the same volume. MYZ028 bacterial liquid was added to the compost at ratios of 1:1, 1:2, and 1:4, with 200 mL added daily. The control group was labeled as DZ, while the labels SY1, SY2, and SY3 corresponded to aerobic composting with bacterial liquid added at ratios of 1:4, 1:2, and 1:1, respectively. Samples were taken at 0 d, 2 d, 4 d, 6 d, 8 d, 10 d, 12 d, and 14 d during the composting process, with three replicates for each group. The samples were used to analyze the physicochemical properties of the compost. After the composting was completed, changes in various indicators in the compost products were analyzed.

### 2.8. The Effect of MYZ028 Concentration on Various Indicators of Compost

The Impact of MYZ028 Concentration on Compost Temperature and pH Value: An electronic thermometer with a range of 100 °C was used to record the temperature of the compost every 2 days during the composting process. For the measurement of the pH value, 5 g of the compost samples was taken and mixed with 100 mL of distilled water in a beaker. After horizontal oscillation at 28 °C and 180 r/min for 1 h, the mixture was left to stand for 30 min; then, the supernatant was taken to directly measure the pH value.

Nitrate and ammonium nitrogen were determined using the colorimetric method. Total organic carbon was measured using the potassium dichromate volumetric method; soluble organic carbon was measured using the deionized water extraction–potassium dichromate–external heating method. Total humic acid was determined using the sodium pyrophosphate extraction–potassium dichromate volumetric method, and free humic acid was determined using the 1% sodium hydroxide extraction–potassium dichromate volumetric method. The fulvic acid and humic acid contents in the compost were determined using the sodium pyrophosphate extraction–sodium hydroxide extraction–potassium dichromate oxidation volumetric method.

## 3. Results

### 3.1. Basic Findings

We obtained diversity data from 12 samples (3 samples each for time points a, b, c, and d) and obtained optimized sequences comprising 21,772,661,012,743,709 bases, with an average sequence length of 465 bp. These results are similar to those of previous research conducted by Xu et al. [9]. The species annotation statistics were as follows: 1 domain, 1 kingdom, 21 phyla, 42 classes, 92 orders, 135 families, 196 genera, 235 species, and 1242 OTUs. The five phyla with the highest abundances were *Proteobacteria*, *Cyanobacteria*, *Firmicutes*, *Actinobacteriota*, and *Bacteroidota*. The genera with the highest levels were *Ralstonia*, *Achromobacter*, *norank_f_norank_o_Chloroplast*, *Pseudomonas*, and *Cupriavidus*. These results are similar to those of research conducted by Chen et al. [1].

### 3.2. Analysis of Microbial Community Composition

The abundance and proportion of different species in microbial communities affect the function and stability of ecosystems. By analyzing the composition of microbial communities, we can understand the interactions between different microbial species.

### 3.3. Venn Diagram of Species

Venn plots were used to identify the numbers of common and unique species (such as ASVs) in multiple groups or samples. This type of analysis intuitively represents the similarity and overlap of species in environmental samples.

**Figure 1:**
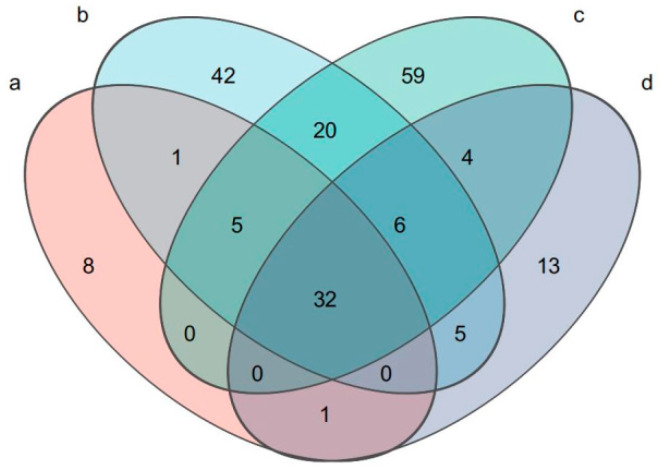
Venn diagram.

In the Figure 1, Sample c contained the most species, along with sample b, and the intersection of samples b and c contained the highest number of species, indicating that there were more common species. However, samples a and d had fewer species than the other sample groups, and the intersection of samples a and d also contained fewer species than the other sample groups. There were fewer common species, indicating that the microbial community structure of the fifth-instar *A. pernyi* larvae was similar from days 2 to 5. This was related to the peak period of the oak silkworm on days 2 and 5, when the microbial species should be related to the food in the intestine.

### 3.4. Community Bar Chart Analysis

**Figure 2:**
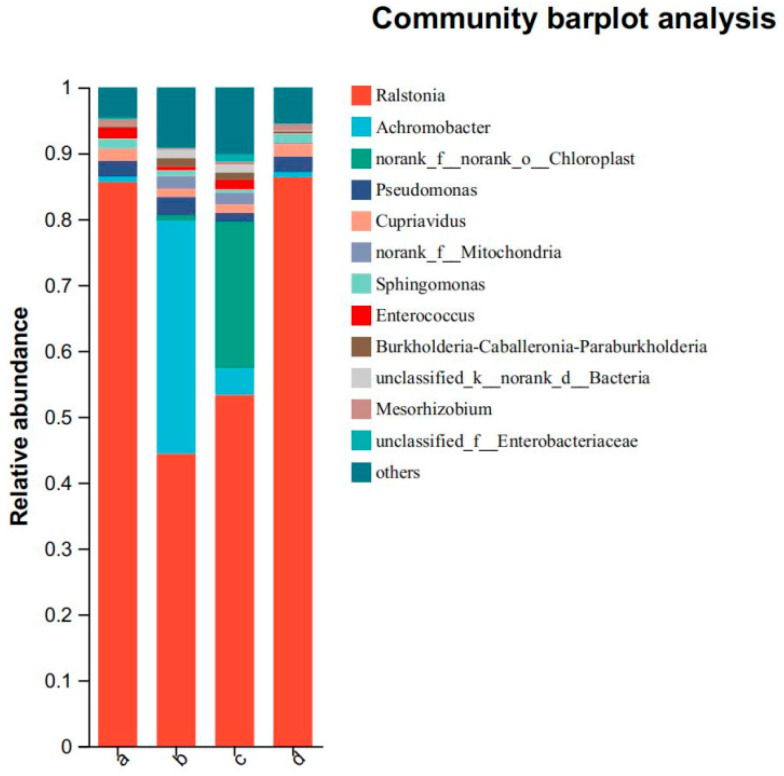
Community bar chart. Note: The horizontal axis represents groups a, b, c, and d; the vertical axis represents the relative abundances of bacterial genera in the sample.

The community composition of the microbiota at various taxonomic levels (i.e., phyla, orders, families, genera, species, and ASVs) can be obtained by studying the phylogenetic relationships among the taxa. The community bar chart (Figure 2) reflects two aspects of the community structure: which microbes were contained in each sample at different classification levels and the content (proportion) of each microbe in the sample.

A bar plot of the microbial community is shown in Figure 2. *Ralstonia* accounted for the highest proportion of bacteria in samples a, b, c, and d, while *Achromobacter* accounted for a high proportion in sample b. Gram-negative bacteria comprised a large proportion in each sample, consistent with previous results on insect gut microbiota [10].

The proportion of *Ralstonia* in the gut of fifth-instar *A. pernyi* larvae was the highest at 85% on day 0. By day 2, the proportion had rapidly decreased to 43%. On day 5, the proportion of *Ralstonia* increased to 53%, while on day 8, the proportion of *Achromobacter* reached 85%. On day 0, the proportion of colorless bacteria was only 1%. However, on the second day of the fifth instar, the proportion of colorless bacteria had increased to 33%. On the fifth day, the proportion of colorless bacteria was only 5%. On the eighth day, the proportion of colorless bacteria was even less, having returned to 1%. In addition to the aforementioned genera, *Pseudomonas*, *Cupriavidus*, *norank_f__Mitochondria*, *Sphingomonas*, *Enterococcus*, *unclassified_f__Enterobacteriaceae*, and unclassified bacteria *unclassified_k__norank_d__Bacteria* were present.

The dominant bacterial genera in the gut microbiota of *A. pernyi* from days 0 to 8 were *Ralstonia*, *Achromobacter*, *Pseudomonas*, and *Cupriavidus*, all of which are in the phylum Proteobacteria. *Enterococcus* and *Mesorhizobium* were secondary dominant bacterial genera. Previous studies have shown that these three dominant bacterial genera all come from plants. Therefore, we concluded that the dominant bacterial genera in the fifth-instar *A. pernyi* mostly come from larval food plants [11].

### 3.5. Community Heatmap Analysis

A heatmap uses color gradients to represent data in a 2D matrix or table, and it can be used to display information on the composition of community types. A commonly used clustering method based on the similarity of abundance was used to divide the samples into classes.

**Figure 3:**
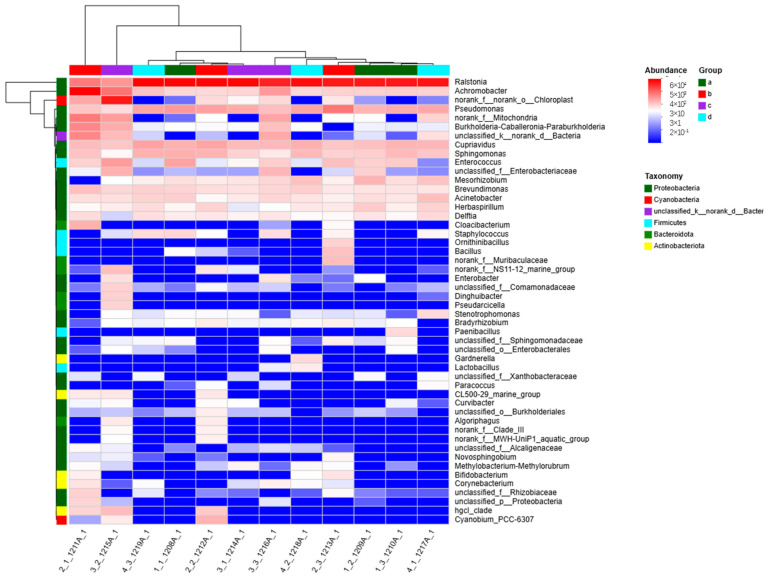
Community heatmap. Note: The horizontal axis represents the sample labels, and the vertical axis represents the species names. A color gradient is used to represent the proportions of species. The value represented by the color gradient is listed on the right side of the graph.

A heatmap (Figure 3) is a commonly used data visualization method to display correlations among data. The heatmap indicated that the genera *Ralstonia* and *Achromobacter* were present in high proportions in each sample. *Ralstonia* belongs to the β-Proteobacteria group in the order Burkholderiales: Burkholderiaceae: Ralstonia of the class of Gram-negative bacteria; *Achromobacter* is sometimes classified as a genus of *Acinetobacter*, also a Gram-negative bacterium. *Acinetobacter* was also included among the four genera of bacteria isolated and identified from the midgut using traditional isolation and cultivation methods.

In addition, except for *Enterococcus,* a Gram-positive bacterium, there was a high proportion of Gram-negative bacteria in each sample. Notably, previous studies have found that the dominant microbial community in the intestines of *A. pernyi* varied among different seasons [12]. Based on the research results of the gut microbiota in *A. pernyi*, it was determined that the factors leading to this result may be differences in oak silkworm varieties, developmental ages, diets, seasons, or temperatures [13,14].

### 3.6. Species Difference Analysis

The median differences between groups were visualized using a bar chart, with the vertical axis representing each group and the horizontal axis representing specific values within the group. The bars represent the median values of each group, with the groups represented by different colors. In addition, colored lines can be used on a bar chart to indicate levels of significance.

**Figure 4:**
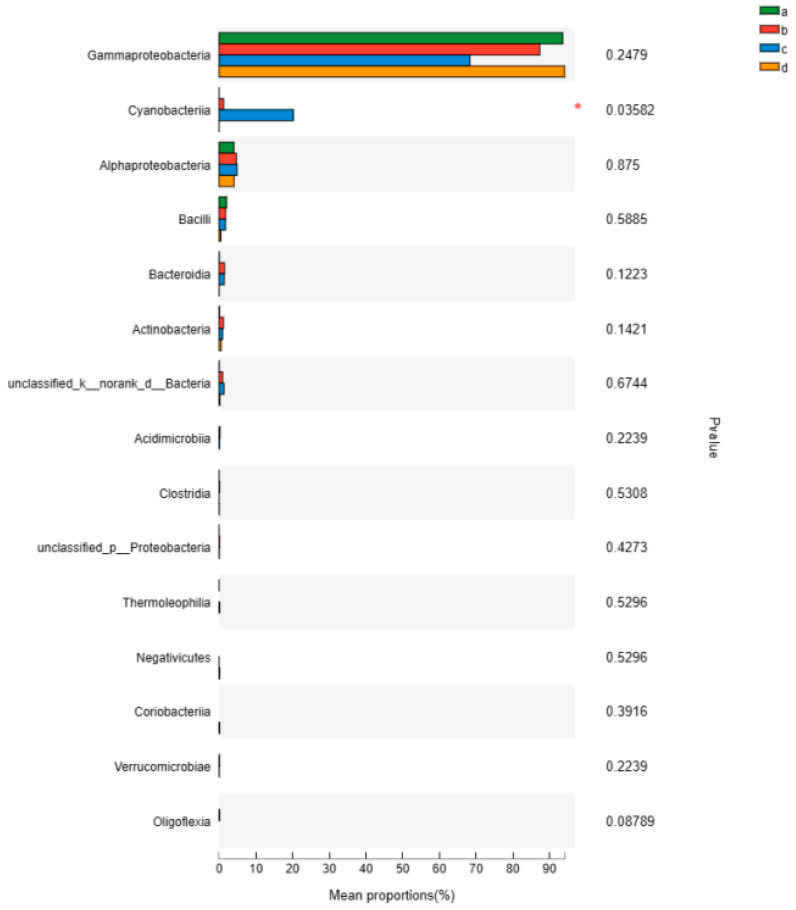
Comparison of multiple groups and single-species groups. Note: The horizontal axis represents the species names at different classification levels; the vertical axis represents the percentage value of a certain species abundance in the sample; and different colors represent different groups. The far right column lists *p*-values; * 0.01 < *p* ≤ 0.05.

Gammaproteobacteria comprised a high proportion in samples a, b, c, and d, while Cyanobacteria only accounted for a high proportion in sample c (Figure 4). The chart shows the significance values for the proportions of each bacterial genus; Alphaproteobacteria had a *p*-value of less than 0.05, suggesting that there were no significant differences in the proportions of other bacterial genera.

**Figure 5:**
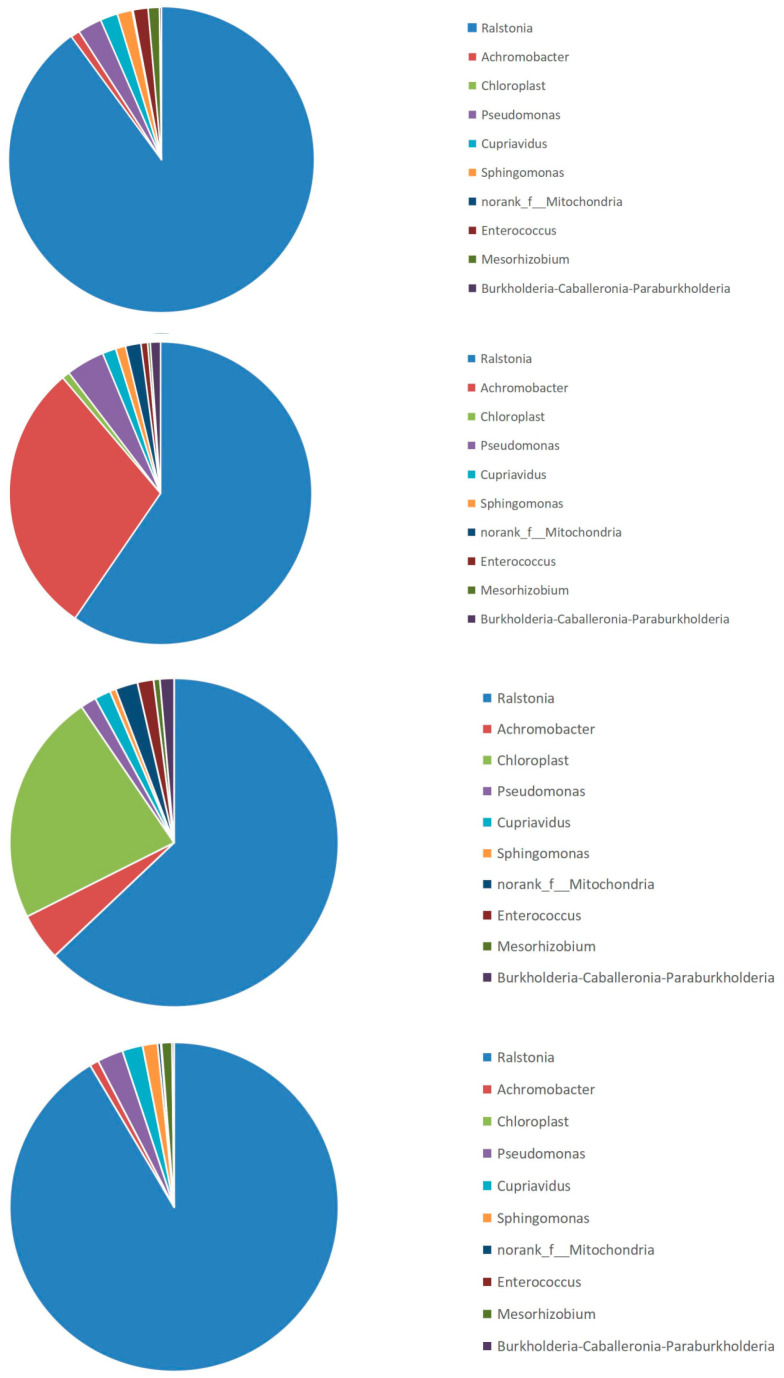
Proportions of each bacterial genus. Note: Groups a, b, c, and d are represented in order from top to bottom.

The pie charts (Figure 5) indicate that the most significant changes occurred in *Ralstonia*, *Achromobacter*, and *Pseudomonas*, suggesting that the gut microbiota was more influenced by diet than by the larvae. This result is consistent with previous findings by Dong Huiling [15].

### 3.7. Degradation Effect of MYZ028 on Straw

The sequencing results were compared with the NCBI database. MYZ028 had a high degree of homology with the sequence of Bacillus subtilis; thus, the strain was identified as Bacillus subtilis, a member of the Bacillus genus.

By measuring the change in the straw mass before and after degradation, the degradation rate of MYZ028 on straw could be obtained. After 14 days of degradation, the straw mass in the experimental group decreased from 2 g to 1.15 g, resulting in a degradation rate of 42.5%. In the control group, the straw mass decreased from 2 g to 1.40 g, with a degradation rate of 30%. Therefore, it was preliminarily concluded that the MYZ028 strain used in this experiment can effectively degrade lignin.

**Figure 6:**
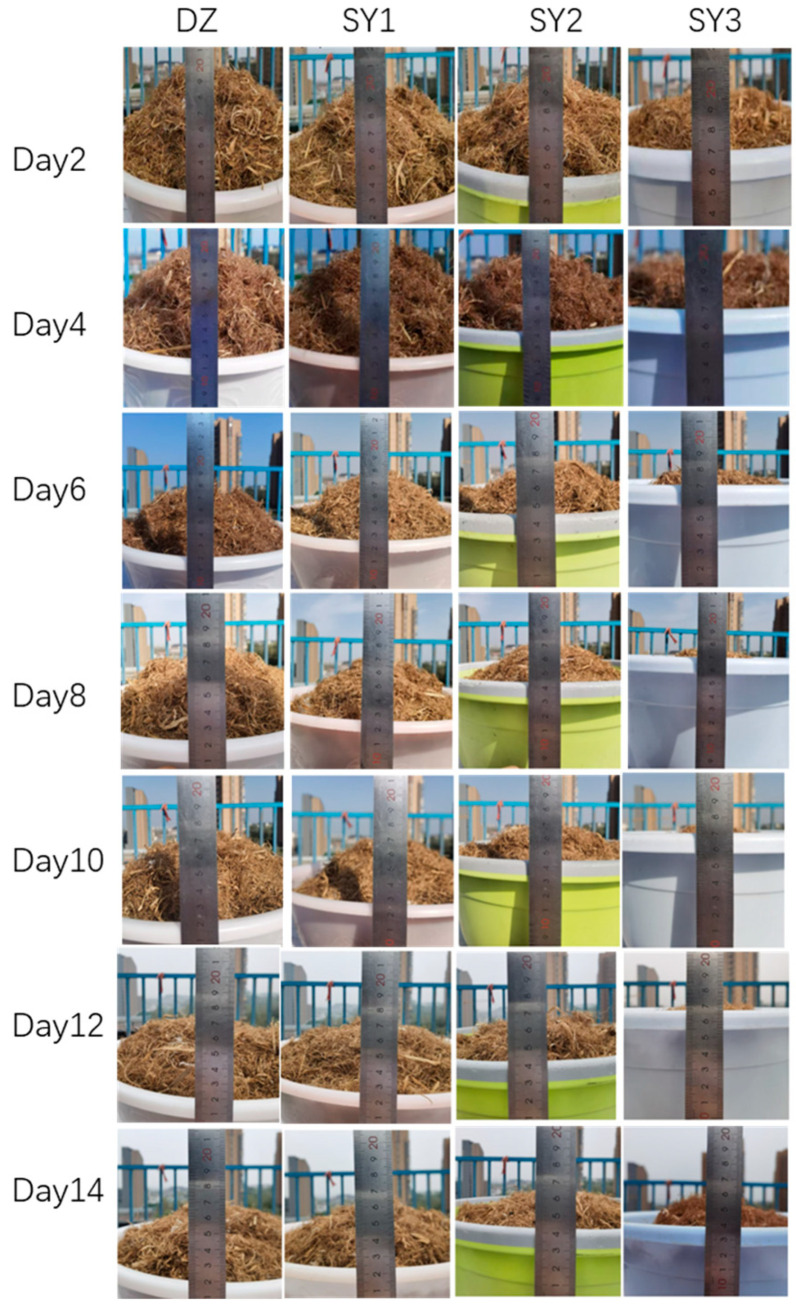
Four groups of composting experiments: height changes from 1 to 14 days.

The results of the 14-day treatment of the four aerobic compost piles are shown in Figure 6. It is evident in the figure that the SY3 experimental group, which was inoculated with MYZ028 at a concentration of 1:1, showed the most significant degradation effect, with the pile subsiding by 5.5 cm. The SY2 experimental group showed a pile subsidence of 4 cm, and the SY1 pile subsided by 2 cm, while the control group showed no significant changes. It can be seen that the addition of different concentrations of the MYZ028 strain had varying degrees of degradation capacity, with higher concentrations resulting in more noticeable degradation effects.

### 3.8. Performance Analysis of MYZ028

MYZ028 could effectively increase the temperature of the reactor, with SY2 with a concentration of 1:2 reaching a maximum temperature of 58 °C, the control group reaching a maximum temperature of 54 °C, SY3 with a concentration of 1:1 reaching a maximum temperature of 56.5 °C, and SY1 with a concentration of 1:4 reaching a maximum temperature of 56 °C. It can be seen that MYZ028 had a significant effect on improving the temperature of the reactor.

MYZ028 also had a certain effect on the pH of the aerobic composting, with SY1 reaching 8.78 at the end of composting, and the pH of the other three groups DZ, SY2, and SY3 being 8.67, 8.7, and 8.68, respectively. It is known that a certain concentration of MYZ028 bacterial solution can increase the pH of the pile.

The effect of MYZ028 on nitrate and ammonia nitrogen during the aerobic composting process showed different results at different concentrations. At the end of the experiment, the nitrate nitrogen content in DZ~SY3 was 0.039, 0.05, 0.045, and 0.041 g/kg, respectively, and the ammonia nitrogen content was 0.69, 0.78, 0.58, and 0.64 g/kg, respectively. It can be inferred that the higher the MYZ028 concentration, the lower the nitrogen content.

MYZ028 had a relatively small impact on the total organic carbon content and soluble organic carbon content during aerobic composting. At the end of composting, the total organic carbon content in each group was around 22%. However, MYZ028 had a certain impact on the reduction ability of the total organic carbon during composting. SY3 sharply decreased from 0 d to 10 d and gradually stabilized, while the decomposition ability of the other three groups was lower than that of SY3. After the experiment, SY3 had the lowest soluble organic carbon content at 6 g/kg, SY2 had the highest at 6.9 g/kg, and DZ and SY1 had values of 6.6 and 6.7 g/kg, respectively.

We examined the effect of MYZ028 on the total and free humic acid contents during the aerobic composting process. During the entire composting period, the total humic acid content in SY3 was higher than that in the other groups. At the end of the experiment, the content was 11.2%, the content in DZ was 10.4%, and the content in both SY1 and SY2 was 10.5%.

MYZ028 had a favorable effect on the fulvic acid and humic acid contents during aerobic composting. The content in DK~SY3 was 14.8, 14.7, 12.1, and 11.8 g/kg, respectively, and the humic acid content was 34.5, 35, 36.7, and 35.2 g/kg, respectively. In summary, different MYZ028 concentrations have a certain promoting effect on various factors of aerobic composting, thus providing a favorable data reference for the green and efficient treatment of crop straw.

**Figure 7:**
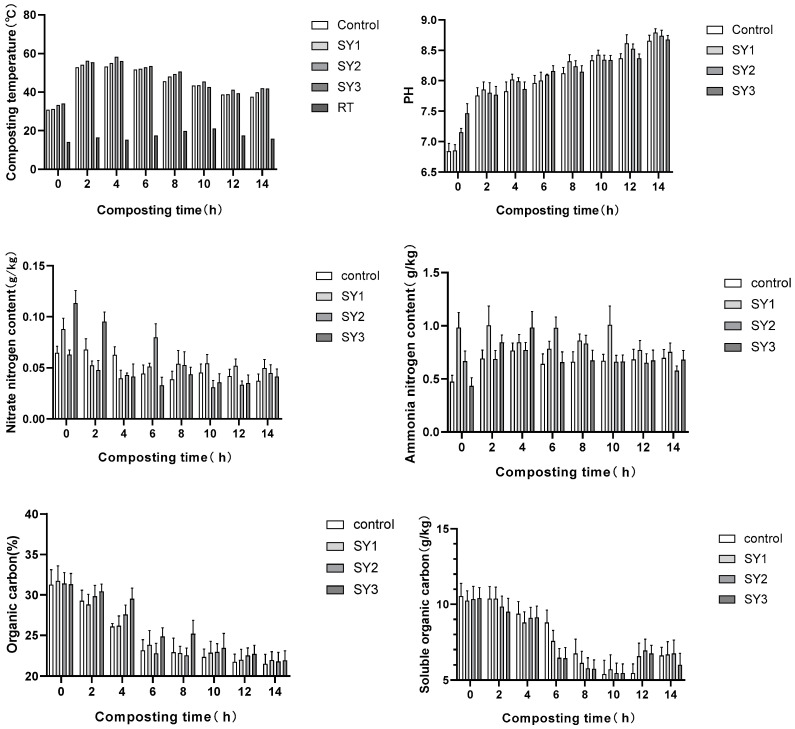

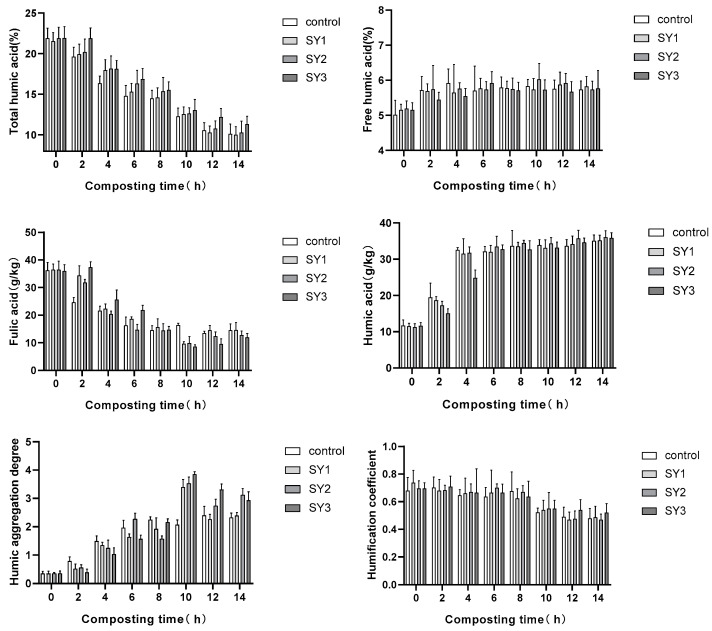
Performance analysis of MYZ028.

The experimental data in this section underwent statistical analysis, and it was found that there was almost no difference in the data between groups (Figure 7).

## 4. Discussion

*Antheraea pernyi* is an insect endemic to China. The moth has a short breeding cycle and is currently one of the most distinctive economically important insects cultivated in China. The species is closely related to various other species, including the domestic silkworm. Previous studies have found that insects contain astonishing numbers of microbes in their intestines and that the abundance of microbiota in most insects increases with development and maturation [16,17,18]. The composition of the gut microbiota varies among silkworm varieties, and the dominant bacterial species varies with developmental age. The microorganisms attached to the surface of mulberry leaves are similar to the dominant gut microbiota of *A. pernyi*, indicating that the microbial composition in the food plant can affect the composition of the gut microbiota [19]. As in *A. pernyi*, the gut microbiota of oak silkworms is closely related to their physiological activities. Bacteria dominate the gut microbiota in insects, accounting for more than 60%; bacteria account for more than 99% of the total gut microbiota of *A. pernyi*. The microbiota can promote the growth and development of *A. pernyi* and improve the quality of the silk from cocoons. The gut microbiota of *A. pernyi* is closely related to its physiological activities, suggesting that the microbiota is important for the growth and development of the moth.

This study used bacterial genomic DNA extraction technology to construct a 16S rDNA clone library of the intestinal microbiota of *A. pernyi*; the library provides a foundation for subsequent research on the relationship between the intestinal microbiota of *A. pernyi* and its physiological activities. The metagenomic sequencing results showed that the proportions of Gram-positive and Gram-negative bacteria in the gut microbiota of the oak silkworms were 25% and 60%, respectively. Among the Gram-negative bacteria, *Proteobacteria* was the dominant phylum. Similar studies found that *Proteobacteria* and *Firmicutes* were the dominant bacterial groups in an analysis of gut bacterial structures in insects in the orders Coleoptera, Isoptera, and Lepidoptera [20]. The microbial communities in the midgut of *A. pernyi* were also dominated by *Firmicutes*, *Proteobacteria*, and *Actinobacteriota*. *Proteobacteria* is present at high levels in insects, animals, humans, and even the air. Researchers have found that some beneficial bacteria in *Proteobacteria* are significant for insect cultivation. For example, in this study, *Achromobacter* and *Ralstonia* belong to the *Proteobacteria* group, and these bacteria could help the silkworm to digest food and inhibit the growth and reproduction of harmful bacteria in its intestines. Therefore, further research on these bacteria is relevant to cultivating oak silkworms to produce strains with high feed conversion rates and increased disease resistance.

Functional research on the gut microbiota of *A. pernyi* showed that the gut microbiota played an important role in regulating the intestinal microenvironment; this can improve the growth rate, accelerate digestive enzyme activity, enhance the immune system, and regulate endocrine function. According to the comparative results of fifth-instar larvae at 0, 2, 5, and 8 days, the gut microbiota is closely related to the growth and development of *A. pernyi*, and it has regulatory physiological functions. *Ralstonia* was the dominant bacterial genus in the four comparison groups. It has been suggested that these *Ralstonia* bacteria originate from the leaf surfaces of *Quercus* and *Morus* trees [21]. *Ralstonia* is often associated with plant pathogenicity and includes plant pathogens that cause serious harm to crops [22]. *Ralstonia solanacearum* causes mulberry wilt disease [23]. It would be valuable to extract and study the dominant genera of bacteria in the gut microbiota of *A. pernyi*, such as *Ralstonia* and *Achromobacter*, and apply them to *Bombyx mori* or other species. Prior studies have used the contents of *A. pernyi* for biological preparations and applied them in *B. mori*, making them potentially valuable in food, silk, and other fields. In future research, we will compare and analyze the results of metagenomic sequencing with those of traditional methods to gain a more comprehensive understanding of the composition, function, and relationship with the physiological activities of the gut microbiota of *A. pernyi*. In addition, further research can be conducted on the mechanism of the gut microbiota of *A. pernyi* in regulating the microenvironment, thereby providing a basis for further research on the gut microbiota of silkworm moths.

We conducted a composting experiment to analyze the degradation performance of MYZ028 and found that different concentrations of MYZ028 could enhance the degradation of lignin during the composting process and had a certain impact on various indicators in the composting experiment. An analysis of the temperature and pH results showed that the higher the MYZ028 concentration, the higher the pile temperature and the lower the pH value. The highest pH value in the experiment was found for SY2 at 8.7, while the pH values for DZ, SY1, and SY3 were 8.67, 8.78, and 8.68, respectively. This result is clearly outside the standard pH range for composting experiments. However, a literature review indicates that during the early stages of compost maturation, the pH value may increase, and in the later stages of maturation, the pH value will decrease to within the standard range. This is because in the early stages of maturation, the pile temperature has not yet dropped below 40 °C, and the microbial activity remains high, continuously breaking down certain acids in the pile, leading to an increased pH value. The addition of MYZ028 bacterial liquid creates a more favorable degradation environment for the microorganisms in the pile, thus promoting the breakdown of large molecules. Furthermore, the exploration of the nitrogen, humic acid, fulvic acid, and humic acid contents revealed that MYZ028 had a certain promoting effect on these factors. In the composting experiment, the overall gain effect of the experimental group SY2 was the most significant, making the optimal MYZ028 concentration ratio in this experiment 1:2. At the same time, it is expected that through genetic engineering and other means, the lignin-degrading ability of this strain can be further improved, making a greater contribution to the effective utilization of lignin resources and environmental protection.

## Data Availability

The original data presented in the study are openly available in BioProject at PRJNA1243856.
